# Dermatological Manifestations of Parkinson's Disease: Clues for Diagnosis

**DOI:** 10.7759/cureus.10836

**Published:** 2020-10-07

**Authors:** Wajeeha Shahid, FNU Satyjeet, Raj Kumari, Kuldeep Raj, Vikash Kumar, Maham Noor Afroz, Muhammad Khizar Memon

**Affiliations:** 1 Internal Medicine, Jinnah Sindh Medical University, Karachi, PAK; 2 Internal Medicine, Chandka Medical College, Larkana, PAK; 3 Internal Medicine, People's University of Medical and Health Sciences for Women, Karachi, PAK; 4 Internal Medicine, Liaquat University of Medical and Health Sciences, Hyderabad, PAK

**Keywords:** parkinson' s disease, dermatological manifestaion

## Abstract

Background and objective

Parkinson’s disease (PD) is a common neurodegenerative disorder. There are various manifestations of PD. Among them, motor dysfunction has been studied in many research studies; however, few studies are available related to the dermatological manifestations of PD. This study was conducted with the aim to shed light on various skin conditions that occur in PD.

Methods

This cross-sectional study was conducted at a tertiary care hospital in Pakistan for a period of nine months; 107 patients with PD were included after obtaining informed consent. A self-administrated questionnaire was used to record demographic data and dermatological findings.

Results

Among the various dermatological manifestations, patients with PD most commonly presented with seborrheic dermatitis (46.7%) and rosacea (10.2%). Other manifestations included bullous pemphigoid (7.4%) and melanoma (4.6%).

Conclusion

The study revealed several dermatological manifestations of PD, which usually get overlooked by neurologists. Through this study, we want to emphasize that PD, apart from all the motor signs and symptoms, can also present as skin problems, and hence, a multi-disciplinary approach should be taken while managing PD.

## Introduction

Parkinson’s disease (PD) is the second most common neurodegenerative disorder [1]. It is caused by the loss of dopaminergic neurons from the substantia nigra [1]. PD is mostly sporadic; however, mutations involving Parkin (PARK2) and PINK1 (PARK6) genes have also been identified [2]. This condition is characterized by the classic triad of rigidity, resting tremors, and bradykinesia [3]. The clinical symptoms of Parkinsonism include stooped posture, festinating gait, masked facies, and dementia.

PD is predominantly a hypokinetic motor disorder, but it may also manifest other non-motor symptoms [4]. The common non-motor symptoms include pain, sweating, seborrhea, insomnia, depression, and cutaneous problems [5]. Despite major evidence supporting the association of neurological disturbances and cutaneous manifestations with PD, the skin problems often get neglected by the doctors [2]. The major dermatological manifestation of PD includes seborrheic dermatitis, melanoma, bullous pemphigoid, and rosacea [2].

Compared to other developing countries such as India and the People’s Republic of China, there is no available information on the prevalence of PD or Parkinsonism from Pakistan. The skin disorder in PD is often overlooked. The accurate diagnosis and treatment of the dermatological problems present in patients with PD can significantly improve their quality of life. This study is conducted to determine the frequency of the dermatological manifestations associated with PD.

## Materials and methods

This study was conducted from April 2019 to December 2019 in the neurology outpatient department (OPD) of a tertiary care hospital in Pakistan. One hundred and seven (107) patients with PD were enrolled after obtaining informed consent. The patients' demographics and characteristics were recorded using a self-administrated questionnaire. Dermatological manifestations were diagnosed with the help of consultations with dermatologists. 

Data analysis was performed with SPSS® Statistics software version 23.0 (IBM, Armonk, NY). Numerical data such as age and mean duration of diagnosis were expressed as means ±standard deviations (SD). Categorical data such as dermatological findings were presented as frequencies and percentages.

## Results

The mean age of the participants was 61 ±14 years. The average time duration for which participants had the disease was 3 ±1 year. The most common dermatological condition was seborrheic dermatitis (46.7%), followed by rosacea (10.2%) (Table 1).

**Table 1:** Dermatological manifestation in patients with Parkinson's disease

Dermatological manifestation	N (%)
Seborrheic dermatitis	50 (46.7%)
Rosacea	11 (10.2%)
Bullous pemphigoid	08 (7.4%)
Melanoma	05 (4.6%)
Other lesions	03 (2.8%)

## Discussion

PD is one of the most common neurodegenerative disorders, and it is characterized by the classic triad of rigidity, resting tremors, and bradykinesia. The disease comprises motor as well as non-motor symptoms. Changes in the skin are common symptoms of PD; however, they are often unrecognized by medical practitioners. In this study, the most commonly reported dermatological manifestation of PD was seborrheic dermatitis, followed by rosacea.

The skin problem in PD is a key determinant of the well-being of the patient. A skin problem often plays a vital role in the low self-esteem of the patient. This study showed a high incidence (46.7%) of seborrheic dermatitis in PD patients. This is comparable to the available data, which shows that the prevalence of seborrhea is as high as 52%-59% in PD [6]. The pathogenesis for the increased prevalence of seborrhea in PD is due to an increase in the production of host sebum as well as the increased production of Malassezia yeast [6]. The association between PD and seborrhea was established as early as 1927, when Krestin found seborrheic facies as a cutaneous manifestation of Parkinsonism [7].

According to our study, rosacea is the second most common inflammatory skin condition. This skin condition is common in PD; it mostly affects the face and plays a key role in negatively affecting the confidence of the patient [4]. The psychosocial impact worsens the condition and compromises the quality of life of the patient. Similarly, a survey conducted by Rainer et al. showed possible treatment options for temporary suppression of symptoms but no possible cure for the condition, leading to mental discomfort among patients [8]. Another study has also established the possible psychosocial impact of rosacea, confirming the major possibility of low self-esteem and distress among the patients [9].

In this study, bullous pemphigoid was found in 7.4% of patients with PD. Bullous pemphigoid is an autoimmune disease and involves the formation of autoantibodies against the skin. However, the exact association between bullous pemphigoid and PD is not well established [10]. Cordel et al. observed that 9% of PD patients had bullous pemphigoid [11]. Another case-control study found a significantly higher prevalence of PD in the case group (patients with bullous pemphigoid) compared to the control group [10].

Melanoma was also found in 4.6% of the patients with PD in this study. One plausible explanation for the potential association between melanoma and PD is that α-Synuclein, an enzyme that inhibits tyrosine hydroxylase, is involved in the melanin synthesis in both melanoma and dopaminergic neuronal cells in PD [12]. Bertoni et al. have found that there is a two-fold higher melanoma prevalence in patients with PD compared to the general population [13]. However, Liu et al., in their meta-analysis, have found that there is only a moderate association between PD and melanoma [14].

To the best of our knowledge, this is the first study conducted in Pakistan that explains the dermatological manifestations of PD. However, this study has its own limitations. This study was conducted in one center only, and this may have limited the diversity of the sample. We recommend that multi-centric studies with large sample sizes be conducted. Another limitation is that since this was a cross-sectional study, the causal relationship between dermatological presentation and PD could not be established. The findings of the studies as recommended by us will help the neurologists to recognize the dermatological signs and symptoms of PD for the better management of the condition.

## Conclusions

In our study, seborrheic dermatitis, rosacea, and bullous pemphigoid were the most common dermatological findings in patients with PD. The dermatological manifestations of PD are often missed or ignored by the treating physicians. Recognizing the dermatological signs and symptoms, along with a better understanding of the possible pathophysiology of the disease, will help improve the quality of life of the patients. It is important that a multi-disciplinary team should take care of PD patients, including the management of dermatological manifestations.
